# Using Climate to Explain and Predict West Nile Virus Risk in Nebraska

**DOI:** 10.1029/2020GH000244

**Published:** 2020-08-27

**Authors:** Kelly Helm Smith, Andrew J. Tyre, Jeff Hamik, Michael J. Hayes, Yuzhen Zhou, Li Dai

**Affiliations:** ^1^ National Drought Mitigation Center, School of Natural Resources University of Nebraska‐Lincoln Lincoln NE USA; ^2^ School of Natural Resources University of Nebraska‐Lincoln Lincoln NE USA; ^3^ Department of Educational Psychology University of Nebraska‐Lincoln; Nebraska Department of Health and Human Services Lincoln NE USA; ^4^ Department of Statistics University of Nebraska‐Lincoln Lincoln NE USA

**Keywords:** disease vector, West Nile Virus, drought, ecologic modeling, prediction

## Abstract

We used monthly precipitation and temperature data to give early warning of years with higher West Nile Virus (WNV) risk in Nebraska. We used generalized additive models with a negative binomial distribution and smoothing curves to identify combinations of extremes and timing that had the most influence, experimenting with all combinations of temperature and drought data, lagged by 12, 18, 24, 30, and 36 months. We fit models on data from 2002 through 2011, used Akaike's Information Criterion (AIC) to select the best‐fitting model, and used 2012 as out‐of‐sample data for prediction, and repeated this process for each successive year, ending with fitting models on 2002–2017 data and using 2018 for out‐of‐sample prediction. We found that warm temperatures and a dry year preceded by a wet year were the strongest predictors of cases of WNV. Our models did significantly better than random chance and better than an annual persistence naïve model at predicting which counties would have cases. Exploring different scenarios, the model predicted that without drought, there would have been 26% fewer cases of WNV in Nebraska through 2018; without warm temperatures, 29% fewer; and with neither drought nor warmth, 45% fewer. This method for assessing the influence of different combinations of extremes at different time intervals is likely applicable to diseases other than West Nile, and to other annual outcome variables such as crop yield.

## Key Points

- A dry year preceded by a wet year in combination with warm temperatures increases the number of human cases of West Nile Virus in Nebraska
- We found that drought accounted for 26% of WNV cases 2002–2018, and drought and temperature together accounted for 45% of cases

## 1 Introduction

**Figure 1:**
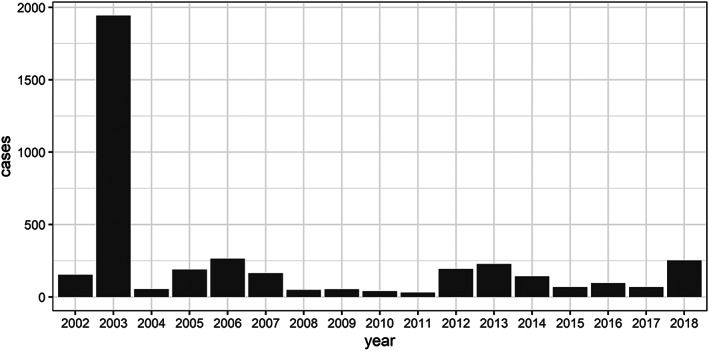
Human cases of WNV in Nebraska, 2002–2018. This figure shows the annual totals of all human cases of West Nile Virus in Nebraska, combining neuroinvasive and non‐neuroinvasive cases. After 2003, when WNV spread through a previously unexposed population and infected more than 1,900 people, annual totals have fluctuated in a smaller range, not exceeding 300.

**Figure 2:**
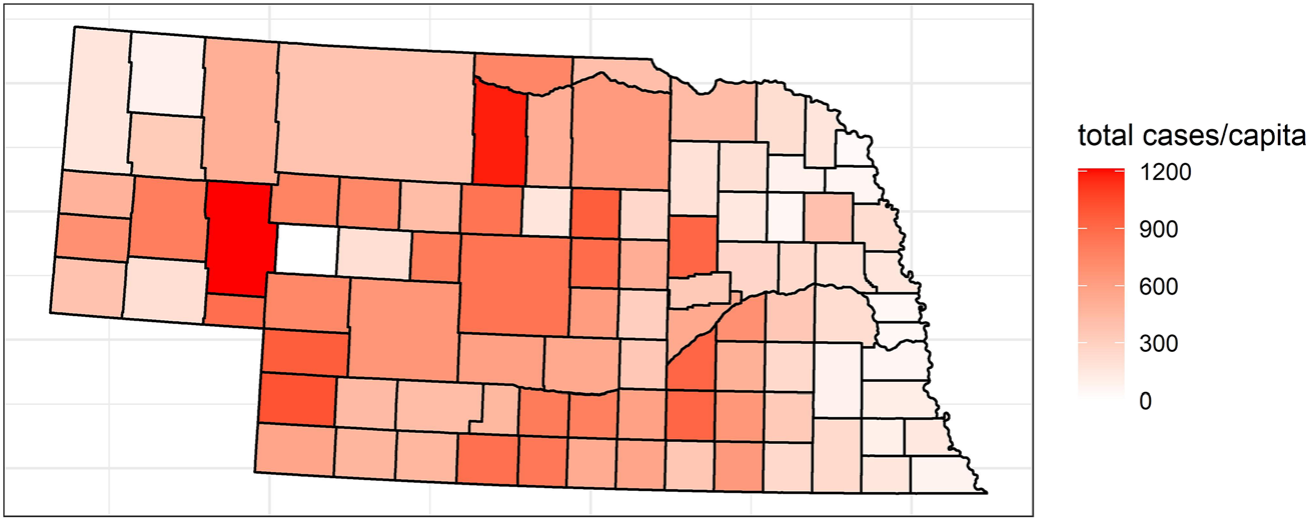
Cumulative incidence of human cases of WNV through 2018. This map of cumulative incidence shows that counties in eastern Nebraska have generally had a lower infection rate. Cumulative incidence is all cases over time, both neuro‐ and non‐neuroinvasive, per 100,000 population.

West Nile Virus (WNV), which is usually transmitted to humans by the bite of an infected mosquito (the vector), made its first documented appearance in the continental United States in New York City in 1999 (Marfin & Gubler, 2001), and it has since spread nationwide, with substantial variation in infection rates from place to place and from year to year. The first cases of WNV in Nebraska were reported in 2002, and in 2003, it spread through a previously unexposed population in Nebraska, with 1,942 cases reported that year, far more than the next‐highest annual total, 264 in 2006 (Figure 1). Counties in roughly the eastern quarter of the state have experienced a lower cumulative incidence rate than the rest of the state (Figure 2), although Nebraska and other Plains states have a relatively high per‐capita infection rate of WNV infection compared with the rest of the country (https://www.cdc.gov/westnile/statsmaps/cumMapsData.html).

An estimated 1% or fewer of those infected develop neuroinvasive forms of WNV, which is occasionally fatal (Mostashari et al., 2001; Petersen et al., 2013). Milder cases of WNV in humans include a fever, or are completely asymptomatic, and the majority of human infections go unreported (Colpitts et al., 2012; Curren et al., 2018; Marfin & Gubler, 2001; Mostashari et al., 2001; Petersen et al., 2013). Whether or not they have symptoms when they are first exposed, people are presumed to have acquired immunity after that (Busch et al., 2008; Rossi et al., 2010; Samuel & Diamond, 2006), and do not have symptoms a second time. The infection season generally runs through summer and fall (CDC, 2013), when virus‐carrying mosquitos feed on humans (Kilpatrick et al., 2006).

Although *Culex tarsalis*, the main WNV vector in Nebraska, breeds in standing water, anecdotal observations, large‐scale statistical analyses, and research in other parts of the country suggest a connection between drought years and higher infection rates (Epstein & Defilippo, 2001; Shaman et al., 2005), perhaps related to irrigation (Petersen et al., 2013), to greater concentration of host species around limited water sources (Brown et al., 2014), or to increased blood‐feeding by thirsty, infected mosquitoes (Hagan et al., 2018). In 2012, a hot, dry year, the state saw a resurgence of WNV (Figure 1), despite lower populations of mosquitoes.

Our goals were (1) explanatory, to see whether we could account for some portion of WNV infections in humans based on precipitation and temperature, and (2) predictive, to see whether we could produce annual county‐level predictions at the start of the infection season, based on temperature, precipitation, and other readily available data, to provide an early warning of years with greater risk of WNV infection. This would complement the in‐season information available from the state's vector‐borne disease surveillance. Nebraska's vector‐borne disease surveillance program, which conducts mosquito trapping in about 30 of Nebraska's 93 counties, yields information on the prevalence of mosquito species and infection rates with roughly a 2‐week interval between data collection and dissemination. The ability to alert practitioners to the potential for higher infection rates early in the season provides more opportunity for mitigation actions such as spraying insecticide or reminding people to use repellant, and for anticipating diagnostic and treatment needs and costs. The Nebraska Department of Health and Human Services issues warnings when the virus is first detected each year and before summer holidays that traditionally involve outdoor activities. Being able to predict outbreaks with more confidence would enable public health authorities to conduct targeted preventive messaging (Davis et al., 2017).

Our project incorporates and builds on findings from other multidisciplinary efforts to understand and predict health‐related effects of climate change on vector‐borne disease, focusing on county‐scale in Nebraska. Mills et al. (2010) created a research plan for climate change and vector‐borne disease and recommended collaborative, multidisciplinary partnerships that take an ecosystem approach, incorporating the effects of climate on the physical environment, and on pathogens, vectors, and hosts, replicated over space and time to understand local variation.

## 2 Literature Review

Previous U.S. research has generally found that temperatures within a certain range are associated with the spread of West Nile (Paull et al., 2017) and that winter temperatures may be particularly influential in the northern Plains (Wimberly et al., 2014), but the relationship to precipitation is complex. Depending on factors such as land use and land cover, climate, and prevalence of various animal hosts and mosquito vectors, precipitation has been found to have positive, negative, unimodal, or undetectable correlations with West Nile incidence, and the influence of different drivers varies spatially (Centers for Disease Control and Chuang et al., 2011; Hahn et al., 2015; Paull et al., 2017; Prevention, 2013; Shand et al., 2016; Wimberly et al., 2008, 2014). California, for example, monitors temperature, precipitation, species abundance, infection rate in mosquitoes, sentinel chickens, dead birds, and human cases and cites predictive capabilities based in part on temperature and drought in the months preceding the infection season (California Department of Public Health, 2019). South Dakota State University has conducted extensive research on WNV in the northern Great Plains, combining remotely sensed weather data with near real‐time mosquito monitoring and infection data, and provides alerts and warnings through its Mosquito Information System (http://mosquito.sdstate.edu/).

A recent large‐scale study across the United States identified the role of different predictors of WNV infection rates across the United States, emphasizing the role of immunity from previous exposure (Paull et al., 2017). They used monthly bias‐corrected precipitation and temperature data and the Palmer Drought Severity Index and computed variables reflecting optimal breeding temperatures and freezes. Using a generalized linear mixed effects model, they found that the importance of predictors varied across the country, and that the model was most accurate where drought and immunity were significant predictors, which was the case in Nebraska. It also examined 15 counties in Colorado, using a generalized linear model with negative binomial distribution, to evaluate the contribution of mosquito data, and found that drought was associated with increased infection rates in mosquitoes.

Another recent large‐scale study calculated z‐scores for each U.S. county to express the number of cases of neuro‐invasive WNV as a number of standard deviations above or below the mean (Hahn et al., 2015). Standardization enabled comparisons across different populations, and limiting it to neuroinvasive cases ensured a more consistently reported subset of cases. They used North American Land Data Assimilation System data for precipitation and temperature, aggregated to county level by averaging the grid cells in each county. They used average annual precipitation and temperature calculations for each county, as well as quarterly temperature and precipitation variables. At the national level, they found a strong positive correlation between temperature anomalies and disease incidence, and no significant relationship between precipitation and infection. At county level, temperature increased likelihood of infections in three of 10 regions of the country, and winter temperature was the most important seasonal predictor. The influence of precipitation and temperature varied by region.

Keyel et al. (2019) used random forest machine learning to identify most important variables in predicting WNV infection rates in New York and Connecticut over various spatial and temporal scales. They compared 66 climate‐related and 20–21 nonclimatic variables and found that climate variables improved prediction of mosquito and human infection rates at county scale.

Shand et al. (2016) developed a model to predict West Nile infection risk for DuPage County, Illinois, based on weekly precipitation, temperature, and mosquito surveillance data. For weather data, they averaged values from two observation stations in the county and accounted for historic norms. They investigated the effects of precipitation and computed “degree weeks” for several different weekly periods on mosquito infection rate (MIR), the dependent variable. They found significant interaction between precipitation and temperature, that lower‐than‐average precipitation made warmer conditions a stronger predictor, and that a dry third quarter was a predictor of higher MIR in the following year's infection season.

Davis et al. (2017) observed that most disease models are retrospective, and called for modeling efforts that make predictions on out‐of‐sample data. Accordingly, they used logistic regression to predict the weekly probability of WNV occurrence at county level, based on 6‐month lags of precipitation and temperature and a mosquito infection index. The model correctly predicted an early onset to the infection season in 2016, based on warm temperatures. The researchers used human infection rates from 2004 through 2015, because 2002 and 2003 were highly atypical, with very high infection rates caused by the virus being introduced to a population with little prior immunity. They also had access to weekly data from the state Department of Health through a special data‐sharing agreement. Most weekly county infection numbers were zero, so the researchers used presence‐absence reporting rather than counts. They used the gridded North American Land Data Assimilation System and sampled the county centroid for precipitation and temperature. Time lags of temperature and moisture data helped predict seasonal patterns (Davis et al., 2018). Also in South Dakota, Hess et al. (2018) used machine learning to identify interannual humidity, temperature, and surface water availability along with land use as key influences on the risk of WNV to humans.

The spread of WNV has been associated with warmer temperatures in higher latitudes, especially warmer winters that are no longer cold enough to halt the viral life cycle, although this dynamic is complex (Beard et al., 2016; Wimberly et al., 2014). While the risk of human exposure in places such as Nebraska will likely continue to change, excessively hot temperatures could limit viral reproduction (Beard et al., 2016).

Scientists' ability to predict or forecast disease is still evolving, as is decision‐makers' ability to use advanced warning for risk communication, anticipating treatment needs, or estimating impacts of interventions (Johansson et al., 2019). Researchers have called for more systematic integration of scientific research with public health decision‐making (Barker, 2019). Surveying WNV modeling efforts to date, Barker grouped models by the type of information they provide: spatial patterns; early warning based on spatiotemporal analysis; and early detection, incorporating surveillance data.

Our approach specifically focuses on how to match timescales for drivers and response variables in complex processes such as incidence of vector‐borne disease, which depend on how life cycles of pathogens and vectors intersect with host species. A review of the state of knowledge and needed research on climate change and vector‐borne disease identifies the need for attention to timescales that effectively couple processes across systems, such as climate and WNV infection (Parham et al., 2015). Our model uses meteorological data and past human cases to provide early warning of higher‐risk years, by county, in the months leading up to the infection season. Rather than using preidentified lags of key variables, our method uses regression models with distributed lags of weather variables (Teller et al., 2016) to identify patterns of precipitation and temperature over time that have the largest effect, and then uses models fit on training data to predict human infection in subsequent out‐of‐sample years. Distributed lags of monthly weather variables predicted variation in plant demography as functions of drought indices for a perennial wildflower (Tenhumberg et al., 2018). Distributed lags of daily meteorological variables predicted seasonal patterns of WNV infection in South Dakota counties (Davis et al., 2018), whereas we use up to 3 years of distributed lags of monthly weather and climate variables to predict annual variations in human cases.

## 3 Methods

### 3.1 Data

Most of the data we used are publicly accessible. We used annual counts and estimates of Nebraska county populations from the U.S. Census Bureau. We obtained annual counts of human cases of WNV for each county from CDC's Arbonet for 2002–2018. Paull et al. (2017) demonstrated that previous exposure to WNV reduces human infection rates, so we computed the rate of cumulative incidence as the total number of previous cases, for each county and each year, per 100,000 population. For precipitation and temperature data, we downloaded monthly values from the National Centers for Environmental Information, National Climatic Data Center (https://data.nodc.noaa.gov/cgi‐bin/iso?id=gov.noaa.ncdc: C00005, Vose et al., 2014). For the Standardized Precipitation Index (SPI) and Standardized Precipitation and Evapotranspiration Index (SPEI) by county, we extracted monthly values from Westwide Drought Tracker netcdf files (Abatzoglou et al., 2017). Although the Palmer Drought Severity Index was also available, we did not use it because of its built‐in time lag (Guttman, 1998).

### 3.2 Statistical Modeling

For each target out‐of‐sample year from 2012 through 2018, we used previous years as training data. For each out‐of‐sample year we used two different training data sets. We started training data sets in either 2002, when cases were first recorded, or in 2004, to omit 2003, the highly anomalous year when WNV first widely spread through Nebraska. We used the R package mgcv to fit generalized additive models with thin‐plate splines for nonparametric modeling of distributed lags of drought and temperature data, using restricted maximum likelihood estimation with a log link and negative binomial distribution (Wood, 2011). Natural‐log‐transformed population was used as an offset variable to directly model cases per 100,000 people.

If there is something unique about a county or year that is not reflected in the covariates, then that county or year could have consistently higher or lower cases than expected. This intraclass correlation can occur whenever a sample unit is measured repeatedly, as we do with both counties (multiple years) and years (many counties) (Zuur et al., 2007). One approach to account for this correlation is to include random effects, coefficients specific to a unit that are assumed to come from a specific distribution (usually normal) with mean zero. Including random effects increases the computational complexity of a model, so as an alternative we estimated categorical fixed effects for county and year using sum‐to‐zero contrasts (also called effects coding). Using sum‐to‐zero contrasts we can interpret the remaining fixed effects as applying to an average county and year. We included county contrasts in all models. We ran models with and without year as a sum‐to‐zero contrast coefficient to ensure that including a fixed effect of year did improve the model.

For each county and year, we created sets of lags of drought and temperature variables, working backward from February. In practice, by mid‐March, data through February are available, and preliminary data on human cases for the previous year are available from Arbonet (at least, that was true in 2018). Mid‐March is potentially early enough to provide time to disseminate and use findings, before the late‐spring start to the infection season. Using February as the start of the lagged data, the February value was lag 0, January was lag 1, December, lag 2, and so on. We experimented with lag lengths of 12, 18, 24, 30, and 36 months and created lags for SPI, SPEI, standardized temperature deviations from the mean, and standardized precipitation deviations from the mean.

Cases were our response variable. We eliminated counties with no cases in any years as outliers so that models would converge. There were three counties with no cases through 2012, dropping to one by 2018. Our global model (1) was

(1) $$\begin{array}{l} \ln\left(\lambda_{i}{,}_{t}\right)=\beta_{0}+f_{1}\left({\text{temp}}_{i,t,m}\right)+\;f_{2}\left({\text{drought}}_{i,t,m}\right)\;+\beta_{1}\left({\mathrm{CI}}_{i,t}\right)+\beta_{2i}+\beta_{3t}+\ln\left(\frac{{\text{population}}_{i,t}}{100,000}\right)\\ y_{i,t}\sim \text{NegBinom}\left(\lambda_{i}{,}_{t},k\right) \end{array},$$

where *i* = county of observation, *t* = year of observation, and *m* = months of lagged observations leading up to the start of the infection season, *β*_1_ is the coefficient for cumulative incidence, and *β*_2_ and *β*_3_ are vectors of sum‐to‐zero contrast coefficients to help account for unique spatial (county) and temporal (year) characteristics. *f*_1_ and *f*_2_ are nonlinear coefficients, functional smoothing curves. *β*_0_ is the intercept. λ is the expected rate of infection, and *k* is the overdispersion parameter for the negative binomial distribution.

In the absence of true predictive capabilities, one means of anticipating the number of human cases is by using a naïve model, or what one might reasonably assume in the absence of other calculations (Hyndman & Athanasopoulos, 2018). In this case we adopted an annual persistence naïve model, anticipating that a county would have the same number of cases as the previous year. An annual persistence null model was better for reflecting the substantial variation between counties and years than averaging the number of cases would have been (see statewide temporal variation in Figure 1). The naïve model was simply the prediction that each county would follow a negative binomial distribution with expected number of cases from the preceding year and the same variance as the observations in the preceding year:

(2) $$\begin{array}{c} {\hat{y}}_{i,t_{0}}=y_{i,t_{0}-1}\\ y_{i,t_{0}}\sim \text{NegBinom}\left({\hat{y}}_{i}{,}_{t_{0}},\mathrm{var}\left(y_{i,t_{0}-1}\right)\right) \end{array}$$

where *t*_*0*_ is the out‐of‐sample year.

### 3.3 Model Evaluation

We used AIC to identify the model that best fit the data for seven different out‐of‐sample years and two different time intervals, starting at either 2002 or 2004. For each of the time intervals, we used AIC to identify best‐fit models with and without the year coefficient, yielding four best‐fit models for each of the seven out‐of‐sample years. We used continuous ranked probability scores to compare the forecasting performance of the four fitted models on the out‐of‐sample data. We also rounded numeric predictions to whole numbers to forecast which counties would have one or more cases. We compared the models with each other and with a naïve model. We used these comparisons to make decisions about whether to omit 2002 and 2003 and whether to use the year coefficient.

After resolving these questions, we narrowed the field and further evaluated the practical value of seven fitted models based on how well they predicted the number of cases per county and per year compared with the naïve model and based on how well they predicted which counties would have any cases. Given that the majority of counties in Nebraska from 2002 through 2018 had zero cases, and that dealing with zeros in statistical models poses its own challenges, the ability to anticipate the presence or absence of cases is of value. It is of significance in the real world because it is likely that only the most severe cases of WNV get reported. In other words, if we could issue a warning for a county at the start of the infection season, it could in theory prevent one or two cases that would have gotten reported, as well as many others with less intense symptoms.

### 3.4 Prediction

Using year as a sum‐to‐zero contrast coefficient introduced a complication, in that out‐of‐sample data had no modeled year coefficient. Five of the seven models chosen by AIC starting from 2002 and five of the seven starting from 2004 used the year coefficient as a predictor, with the improvement in AIC scores from adding year generally growing larger as more data were added. To get around this, we modeled a new county‐year (“coyr”) variable for each county: We fit a model using the estimated coefficient for the year as the response variable and the lagged drought and temperature variables and each county coefficient as predictors (Formula 3). Then we used that model to predict a new value for each county‐year (Formula 4). We substituted the predicted county‐year variable for the estimated year‐as‐contrast‐coefficient to predict cases for out‐of‐sample years (Formula 5).

(3) $$\begin{array}{l} \mu_{i,t}=f_{3}\left({\text{temp}}_{i,t,m}\right)+\;f_{4}\left({\text{drought}}_{i,t,m}\right)+\beta_{4i}\\ \;\beta_{i,t}\sim N\left(\mu_{i,t},\sigma^{2}\right) \end{array}$$

where *β*_*i*, *t*_ is the year coefficient at county *i*; *f*_*3*_ and *f*_*4*_ are modeled nonlinear coefficients, functional smoothing curves; and *β*_4*i*_ is a separately estimated county coefficient.

(4) $$b_{3,i,t_{0}}={\hat{f}}_{3}\;\left({\text{temp}}_{i,t_{0},m}\right)+\;{\hat{f}}_{4}\left({\text{drought}}_{i,t_{0},m}\right)+{\hat{\beta }}_{4i},$$

where $b_{3,i,t_{0}}$ is the predicted county‐year coefficient, the variable we called “coyr”; *t*_0_ is the out‐of‐sample year; and ${\hat{f}}_{3},{\hat{f}}_{4}\;\text{and}\;{\hat{\beta }}_{4i}\;$ are the coefficient functions fitted in (3).

(5) $$\begin{array}{l} \ln\left(\lambda_{i}{,}_{t_{0}}\right)=\beta_{0}+f_{1}\left({\text{temp}}_{i,t_{0},m}\right)+\;f_{2}\left({\text{drought}}_{i,t_{0},m}\right)\;+\beta_{1}\left({\mathrm{CI}}_{i,t_{0}}\right)+\beta_{2i}+b_{3,i,t_{0}}+\ln\left(\frac{{\text{population}}_{i,t_{0}}}{100,000}\right)\\ y_{i,t_{0}}\sim \text{NegBinom}\left(\lambda_{i}{,}_{t_{0}},k\right) \end{array}.$$

### 3.5 Evaluating Predictions

In addition to using continuous ranked probability scores to evaluate out‐of‐sample forecast performance, we evaluated predictions for years included in training data, with the goal of identifying patterns that might better inform model selection. For each of the seven models selected, we compared predicted and actual numbers of cases for training data by standardizing them by year and by county and comparing the range and mean of the difference between predicted and actual numbers of cases for both the fitted and naïve models, expressed in standard deviations from the mean. Because 2003 was a year unlike any others, we only compared performance with the naïve model for years after 2004, to avoid unduly handicapping the naïve model.

We paid particular attention to predictions using 2012 and 2018 as out‐of‐sample data, given that those were years that saw comparatively larger changes in the number of cases, and the ability to anticipate a jump in the number of cases would be valuable. We also evaluated the models' ability to predict locations where one or more cases would occur, given that most county‐year combinations had none. We evaluated presence/absence predictions using accuracy, a traditional epidemiological statistic, defined as the total proportion of counties correctly predicted, positive or negative.

### 3.6 Scenarios

To quantify the effect of drought and temperature on WNV in Nebraska, we fit models based on what we knew through February of 2019, including preliminary human cases for 2018. We predicted how many cases there would have been without drought by changing all negative drought index values to zero, effectively eliminating drought before predicting. We did the same with positive values of standardized, mean‐centered temperature, and then with both drought and temperature.

## 4 Results

### 4.1 Comparison of Modeling Choices

Table 1 provides key points of comparison for the four AIC‐chosen models for each of the seven out‐of‐sample years. For the models incorporating data through 2014, 2015, 2016, and 2017, starting from 2002 and from 2004, all eight of the lowest AIC scores were for models with the county‐year coefficient. In three of the six comparisons of earlier years, with less data, AIC scores for models with and without year were within two points of one another. Two of the remaining three had lowest AIC scores for models without the year coefficient, and one included it. Comparing CRPS scores of the 28 fitted models, with and without year, starting from 2002 or from 2004, 17 of the 28 fitted models performed better than the naïve, with almost twice as many, 11 of the 17, fit on all of the data from 2002. Eight of the 17 included the year coefficient. Picking a single lowest CRPS score out of the four for each out‐of‐sample year, four of the seven were fit on data from 2002 and used the year coefficient. Another was fit on data from 2002, was the best choice by AIC, and did not use the year coefficient. The other two were each fit on data from 2004, one with and one without the year coefficient. We examined 2012 and 2018 more closely, years when the numbers of cases increased and would have been particularly valuable to predict. For 2012, all four models out‐performed the naïve, as one would expect. The best‐performing model was fit on data from 2002 and used the year coefficient. For 2018, the model fit on data from 2004 using the year coefficient failed to do better than the naïve model, and the model that did best used data from 2002 and incorporated the year coefficient. Testing residuals from the global model using Moran's I revealed no evidence of spatial autocorrelation. Based on all of the above considerations, we retained the year coefficient as one of the variables to evaluate by AIC for inclusion. We also used all data, from 2002, including the highly anomalous year of 2003, because it resulted in better forecasts.

**Table 1:**
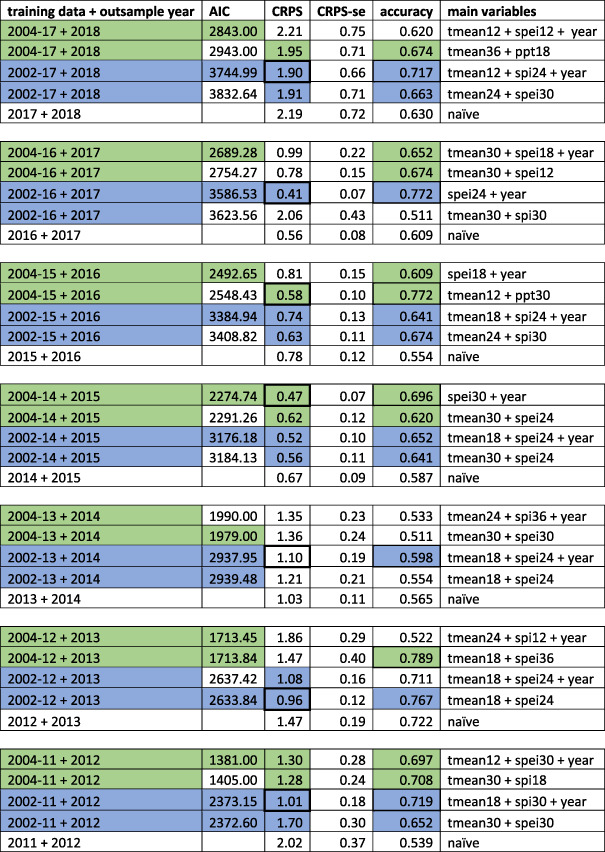
Comparison of Models Using Data From 2002 and From 2004, With and Without Year Coefficient

Table 1demonstrates the rationale for using data from 2002 and for incorporating the year coefficient. The first column describes the years in the training and out‐of‐sample data, with training data starting in 2004 in green and data starting in 2002 in blue. AIC scores contrast models with and without year, and models with lowest AIC scores are shaded green or blue. AIC scores are only used to compare models fit to the same data. Continuous ranked probability scores (CRPS) facilitate comparisons of predictions for each out‐of‐sample year, including comparison with an annual persistence naïve model. CRPS scores for models that out‐perform the naïve are shaded, and the fitted model that did best for each out‐of‐sample year has a thicker border. Accuracy describes the proportion of counties predicted correctly as presence‐absence data in each out‐of‐sample year. All formulas incorporated cumulative incidence, the county contrast variable (random effect), and population as a log offset, so here we only describe the variables and lag lengths that distinguish models from one another.

### 4.2 Exploration of Models

**Figure 3:**
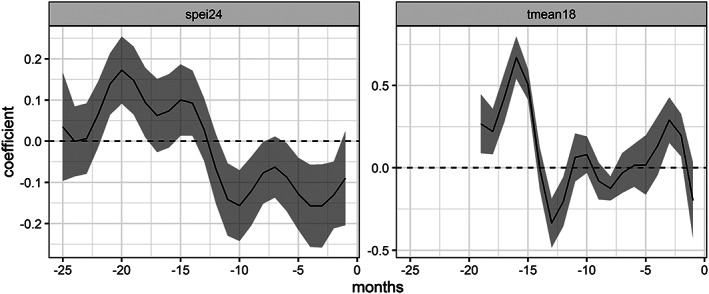
Lagged coefficients for SPEI and mean temperature, model trained on data 2002–2012. The fitted model produced coefficients for each month that showed the influence of drought and temperature variables through February of the year being predicted. Each 1‐unit change in SPEI or mean temperature multiplied by the coefficient, added for each of the months, increased or decreased our response variable, the log of human cases per 100,000 people. A general theme pattern that emerged from many models was that warm temperatures and a dry year preceded by a wet year produced more human cases.

For the sake of expediency, we further describe only the seven models fit on data from 2002 and chosen by AIC, most of which included the year coefficient. Although there was some variation in the lag lengths selected, six out of the seven models used 24 months of a drought indicator, with the other using 30 months, and five out of the seven used SPEI, with the other two using SPI. Notably, one used only SPEI and not temperature. Four of the models used 18 months of temperature, with the others using 12, none, and 30 months. All of the lagged drought coefficients were statistically significant to the 0.01 level or beyond, while four of the temperature lags were only significant at the 0.1 level. Five of the seven models incorporated the year coefficient. The significance of the contrast coefficients for each county varied from no significance to highly significant, with *p* values near zero. As expected, cumulative incidence was a consistently significant predictor, with a negative effect. In other words, the more people that had already had the virus, the fewer unexposed people remained, and the more people were protected by acquired immunity. Variations on a consistent theme emerged from plotting the lagged variables (Figure 3): A dry year preceded by a wet year increased infection rates, as did warmer temperatures. Some models specifically pointed to warmer winter temperatures.

### 4.3 Location Prediction Performance

**Figure 4:**
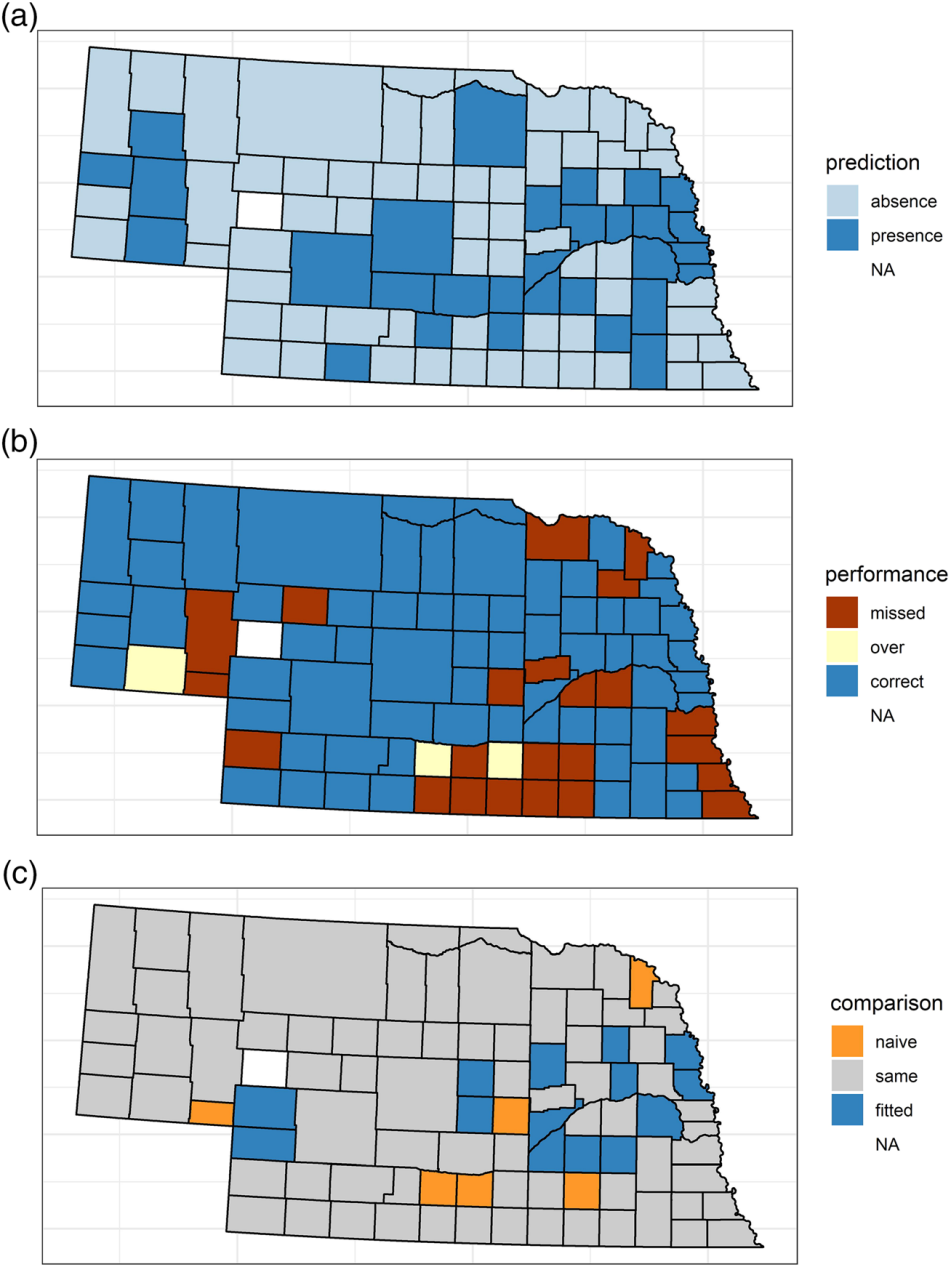
The 2018 presence/absence predictions, performance of predictions, comparison with naïve. (a) Counties predicted to have cases in 2018. “Absence” means counties were predicted to have no cases. “Presence” means they were predicted to have at least one case. Arthur County, white, is “NA” because it has never had any cases and was excluded as an outlier. (b) County prediction performance in 2018. “Missed” means one or more cases occurred in a county not predicted to have cases. “Over” means one or more cases were predicted but did not occur. “Correct” means that a prediction for either the presence or absence of cases was correct. (c) Performance of fitted vs. naïve model in predicting counties with cases. Counties in orange are ones that the naïve model got right but the fitted model did not. Counties in blue are ones that the fitted model got right and the naïve did not. The models performed the same, right or wrong, in counties that are gray.

Fitted models generally did better than the naïve in predicting which county‐years would have one or more cases (Figures 4a–4c; Table 1). In comparisons of fitted and naïve models, we only compared years from 2005 onward, because of the anomalous nature of 2003, which meant that the naïve model would inevitably do worse in 2004. The naïve model was remarkably consistent, with its accuracy for 2005 through 2016 falling in a narrow range from 63% to 65.3%. However, in all cases, the fitted models were more accurate than the naïve model for the years in the training data. The fitted models' accuracy for years 2005 and after ranged from 68.4% to 75.1%. For years from 2002 onward, fitted models' accuracy ranged from 68% to 75.7%.

Our exploration of model selection methods other than lowest AIC did not find relationships between any measures of training data performance and out‐of‐sample performance that led to better results than AIC. On the whole, selection by AIC produced a better set of results than choosing models with or without the year coefficient, or choosing by other model characteristics such as least sum of residuals, or training data performance measures.

To see whether the difference between fitted and naïve models was statistically significant, we performed McNemar's test of paired categorical data. Comparing accuracy of predictions for years in training data from 2005 through 2017, the difference between the fitted and naïve models was statistically significant in all cases. For out‐of‐sample forecasts, accuracy was higher in all cases for the fitted than for the naïve model, for models chosen by AIC and based on data through 2002. Combining all of the out‐of‐sample predictions across years and conducting McNemar's test yielded a statistically significant difference between predictions from the fitted and naïve models, with a chi‐square statistic of 10.97 on 1 degree of freedom, for a probability of 0.0009.

### 4.4 Numeric Prediction Performance

**Figure 5:**
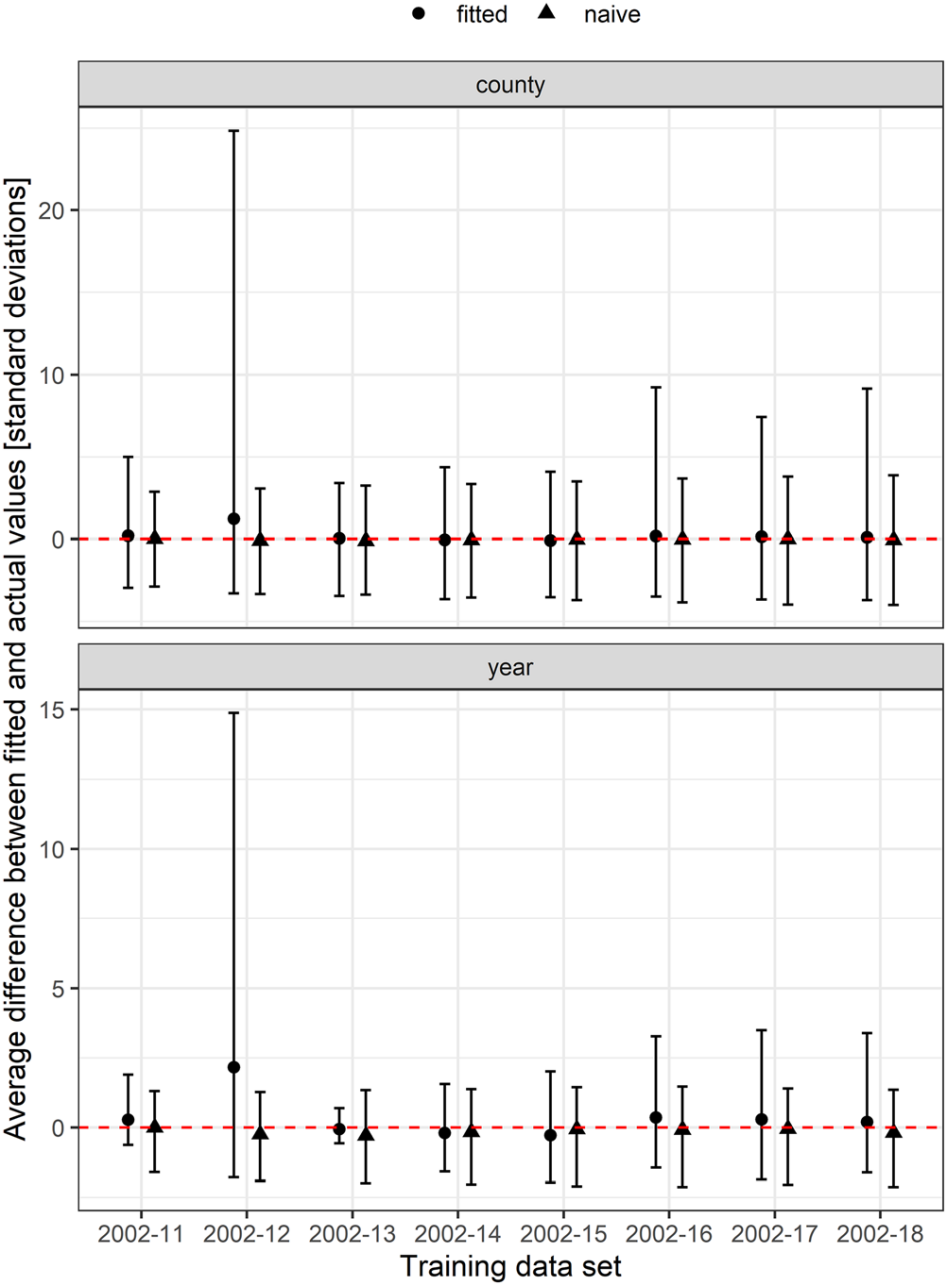
Numeric prediction performance comparisons. This figure contrasts the performance of best fitted models for each set of training data with the performance of an annual persistence naïve model, comparing predictions with actual cases from 2005 on (to avoid handicapping the naïve). The difference between predicted and actual cases is grouped by year and by county and expressed as standard deviations, with an icon indicating the mean difference. Intervals show the minimum and maximum single year or county with the smallest or largest difference between predicted and observed cases.

We compared performance of naïve and fitted models' predictions with actual numbers of cases for years used in training data (starting with 2002–2011, successively adding a year at a time, through 2018), grouped by year and by county, and expressed differences as standard deviations from the mean. We found that the fitted models were more prone to overpredictions, in some cases, dramatically (Figure 5). Compared by year, the naïve model tended to underpredict the number of cases, with the lower boundary of the range smaller in all seven instances than the fitted model, by as little as 0.12 standard deviations for the model fit through 2012, and by as much as 1.43 standard deviations for the model fit through 2013. But the overpredictions of the fitted models were larger than those of the naïve models, in all years but one, and in one case, based on data through 2012, the fitted model's overpredictions were as much as 14.87 standard deviations above actual cases. In all cases but one, based on data through 2013, the average difference between the predicted and actual number of cases was lower for the naïve model than for the fitted model. Grouping and comparing by county told a similar story. The underpredictions of the fitted and naïve models were closer to each other, but the overpredictions of the fitted model were larger, by as much as 24.83 standard deviations for data fit through 2012. Comparing the averages of predictions by the fitted and naïve models, the naïve model came closer on numeric predictions than the fitted models in five out of the seven model runs, with one producing a tie, and the fitted model outperforming the naïve based on data through 2013.

**Figure 6:**
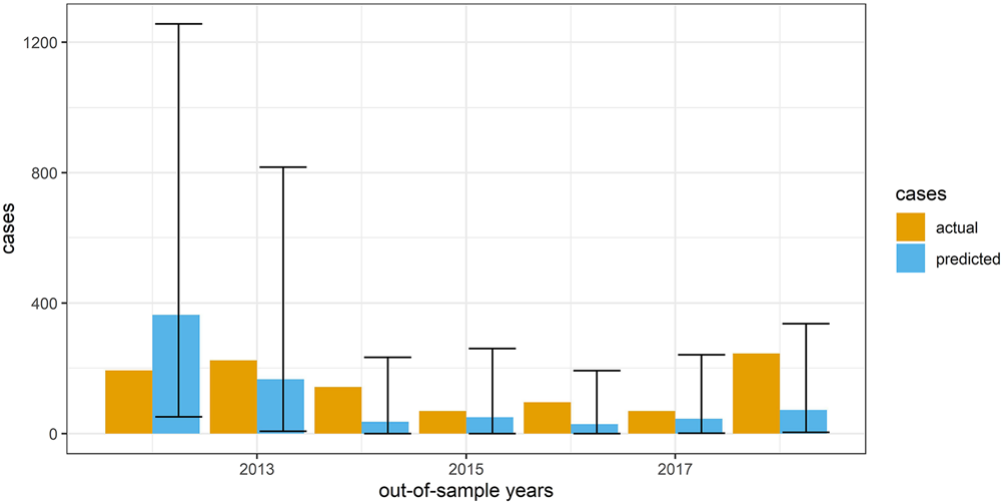
Predicted vs. observed cases, with prediction intervals, for out‐of‐sample years, 2012–2018. This chart compares actual and predicted numbers of cases, with actual numbers in tan, and predicted numbers in blue, with prediction intervals.

The prediction intervals were very large (Figure 6), so even on training data, most but not all of the actual numbers of cases fell between the upper and lower bounds of the prediction interval, but some of the fitted models made dramatic overpredictions for certain years, notably for 2007 with the model fit on data through 2012. The model fit on data through 2011 did a reasonable job of anticipating the increase in cases in 2012, but the model fit on data through 2017 failed to anticipate an increase in cases in 2018. We did not find a sufficiently useful relationship between performance of retroactive predictions and out‐of‐sample forecasts to enable us to make confident numeric predictions. Figure 6 shows predictions for each out‐of‐sample year in blue, with prediction intervals, and the actual number of cases in tan.

### 4.5 Scenario Modeling

**Figure 7:**
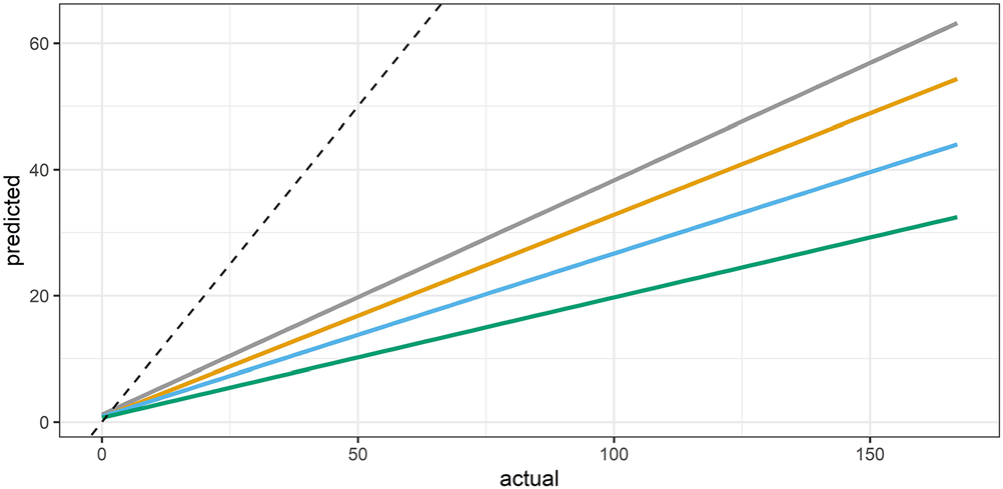
Scenarios without drought, without warm temperature, and with neither. The gray line shows predictions of our fitted model, the baseline scenario. The orange line is the no‐drought scenario. The blue line is the no‐warm temperature scenario. The green line shows the scenario with neither drought nor warm temperatures. The dotted line is where a 1:1 predicted‐to‐actual relationship would be.

How to isolate and quantify the effect of drought—what can we attribute to drought?—is a question that comes up frequently in research on the impacts of drought. We experimented with using a model fitted on data through 2018 to create scenarios without drought, without warm temperatures, and with neither, to quantify the effect drought had on human cases of WNV in Nebraska (Figure 7). Our model fit on data through 2018 predicted 3,318 cases, fewer than the 3,973 actually recorded (excluding 2003, the model predicted 2,467 cases, more than the 2,030 that occurred in years other than 2003). This is our baseline scenario. Our no‐drought scenario estimated that there would have been 2,445 cases of WNV, the orange line, which is 873 less than the baseline scenario, accounting for 26% of the total. Our no‐high temperatures scenario, the blue line, estimated 2,355 cases, 963 less than the baseline scenario, a 29% difference. In the scenario without drought or warm temperatures, the green line, our model predicted 1,835 cases, which is 1,483 less than baseline scenario, and 45% of the total.

## 5 Discussion

We demonstrated that using regression models with distributed lags at monthly intervals can identify patterns of drought and temperature in previous years that predict increased risk of human cases of WNV in Nebraska counties in the subsequent year. Although models fit on training data and used to make out‐of‐sample predictions were better at explanation, i.e., finding the drought and temperature signal in what had happened in the past, they also demonstrated statistically significant early‐season ability to predict where cases would occur, based on out‐of‐sample data, a much more difficult task.

The value of the “year” coefficient, which started as a workaround in the transition from explanatory modeling to forecasting, was an interesting and unexpected finding. Because “year” was a sum‐to‐zero contrast coefficient within the model that we did not have for out‐of‐sample years, we modeled a year coefficient for each county‐year based on the first model, using drought, temperature, and county as predictors, and used that for out‐of‐sample predictions. Other methods such as averaging year coefficients (which we also tried) or setting the year coefficient to zero would lose information about the place‐specific interactions of precipitation and temperature. Although it introduces complexity, the interim step of modeling the year coefficient in effect fine‐tunes the signal for the effects of temperature and precipitation in a specific county. The fact that this workaround produced meaningful results suggests that the interaction of precipitation and temperature matters, as do place‐specific factors such as land use that are not captured in our model. A less complex, more integrated method for fitting smoothing curves for interacting variables would be valuable.

Using Nebraska counties as a unit of analysis also introduced heterogeneity into our model, given that they range from sparsely populated rural areas to dense urban areas. Our model performed better at the “where” question implicit in lower density counties that do not always have cases and worse at the “how many” question implicit in more populous areas that always have cases. This suggests that distinguishing between rural and urban counties, possibly modeling them separately, could improve performance, in lieu of actual access to data at finer spatial scales.

Our model results are consistent with the understanding that drought and temperature contribute to but do not independently account for all cases of WNV in humans. Predictors reflecting additional biological processes could help refine numeric predictions. Population and movement of animal hosts, particularly birds, could prove to be useful predictors (Moon et al., 2019). Population turnover and immunity cycles in avian communities would be valuable to explore as predictors. MIRs would be a logical predictor to add to the model, although only about 30 counties in Nebraska conduct mosquito trapping, and it has been intermittent in some counties, so the resulting data are not evenly distributed across time or space. If real‐time data on human cases were to become available for Nebraska, it would open new avenues for exploration, in combination with drought, temperature, and mosquito data (Davis et al., 2017; Wimberly et al., 2013). The importance of temperatures identified by our models suggests that further research focused on biologic factors influencing whether the virus survives winter months and on earlier mosquito emergence (Ciota et al., 2011) would be valuable. Human behavior is another key influence (Beard et al., 2016), from production‐driven decisions on land use to personal choices about whether to wear protective clothing or insect repellant.

A preliminary use for our predictive maps would be to produce an experimental risk map denoting counties predicted to have higher risk, and use it to underscore a primary message, that people state‐wide should avoid exposure to mosquito bites by emptying pools of standing water, and by wearing protective clothing and insect repellant. Additional warnings could be targeted to counties predicted to have cases, with adequate information about the associated uncertainty of the prediction. We also used the model to create scenarios with no drought conditions and/or no warm winters. A similar process could be used to model the effects of pervasive warmer temperatures. Although the results are fundamentally hypothetical, they would provide sound numeric inputs for scenario‐based decision making, particularly as the practice and use of disease forecasting evolves.

The strength of this method is in using lags of publicly available weather data that can be compared to a response variable to find a meaningful pattern that can be used to explain the past and predict the future. Depending on availability of data, it could easily be expanded to other diseases or other outcomes that are determined partially or wholly by the weather, such as crop yield.

## 6 Conclusion

Human cases of WNV are the result of a complex chain of biological, physical, and social processes, including virus transmission and reproduction through mosquitos and animal hosts such as birds, effects of temperature and precipitation on reproductive success, habitat and feeding behavior, and human choice such as emptying standing water and wearing protective clothing or insect repellant. We found that a dry year preceded by a wet year, and generally warmer temperatures, is a pattern that contributes to higher infection rates in humans in Nebraska, and that a statistical model based on these weather patterns has a statistically significant ability to predict which counties will have cases of WNV. This model allows us to explore scenarios, such as how many fewer cases of WNV there would have been without drought or warm winters. The method we used for detecting drought and temperature signals could be applied to other diseases, crop yield, or other annual response variables.

## Conflict of Interest

The authors declare no financial or institutional conflict of interest related to the research described in this paper.

## Data Availability

Most data are publicly available, as cited in the text and references. Human cases data are visible via CDC's Arbonet disease map, formerly housed at U.S. Geological Survey (https://wwwn.cdc.gov/arbonet/maps/ADB_Diseases_Map/index.html), and researchers can request it from Arbonet via dvbid2@cdc.gov for use. Monthly precipitation and temperature data are from the National Centers for Environmental Information, National Climatic Data Center (https://data.nodc.noaa.gov/cgi‐bin/iso?id=gov.noaa.ncdc:C00005) as described in Vose et al. (2014). Standardized Precipitation Index (SPI) and Standardized Precipitation and Evapotranspiration Index (SPEI) are extracted monthly values by county from Westwide Drought Tracker netcdf files (Abatzoglou et al., 2017). Population data are from the U.S. Census Bureau.
